# The Role of Sphingolipids in the Pathogenesis of Psoriasis

**DOI:** 10.3390/metabo12121171

**Published:** 2022-11-24

**Authors:** Mateusz Matwiejuk, Hanna Mysliwiec, Adrian Chabowski, Iwona Flisiak

**Affiliations:** 1 Department of Dermatology and Venereology, Medical University of Bialystok, 15-540 Bialystok, Poland; 2 Department of Physiology, Medical University of Bialystok, 15-222 Bialystok, Poland

**Keywords:** psoriasis, ceramide, sphingolipid, sphingosine lipid signaling, sphingosine 1-phosphate, sphingosine kinase, sphingomyelin, ceramide S1P receptor

## Abstract

Psoriasis is a complex, chronic, immunologically mediated disease which involves skin and joints. Psoriasis is commonly connected with numerous other diseases such as liver diseases, metabolic syndrome, impaired glucose tolerance, diabetes mellitus, atherosclerosis, hypertension, and ischemic heart disease. Interestingly, comorbidities of psoriasis are an attention-grabbing issue. Additionally, it can cause impairment of quality of life and may be associated with depressive disorders. Altered levels of ceramides in psoriatic skin may lead to anti-apoptotic and pro-proliferative states, consequently leading to an over-proliferation of keratinocytes and the development of skin lesions. The pathophysiology of psoriasis and its comorbidities is not fully understood yet. Sphingolipids (including ceramides) and their disturbed metabolism may be the link between psoriasis and its comorbidities. Overall, the goal of this review was to discuss the role of sphingolipid disturbances in psoriasis and its comorbidities. We searched the PubMed database for relevant articles published before the beginning of May 2022. The systematic review included 65 eligible original articles.

## 1. Introduction

Hannun et al. [1] highlight that the biochemical pathways leading to ceramide generation have been well studied and described in the past. Ceramides can be produced via the catabolism of sphingomyelin and glycosphingolipids, as well as via de novo biosynthesis starting from the formation of sphingoid bases and their subsequent N-acylation [1]. Kozłowska et al. [2] informed us that ceramides play a role in the pathogenesis of psoriasis vulgaris and metabolic disorders such as atherosclerosis, obesity, type 2 diabetes, and cardiovascular diseases. Palmitic ceramide may be involved in the transformation of hepatitis steatosis or obesity-derived insulin resistance [2]. Łuczaj et al. [3] found that the level of prosaposin, which is an essential cofactor in the hydrolysis of sphingolipids, is much lower in psoriatic plaques compared to non-lesional psoriatic skin [3]. Kozłowska et al. [2] revealed that psoriasis is an chronic, inflammatory, immunologically mediated disease which affects not only skin, but also knee joints, fingernails, etc. Psoriasis can systematically recurs or flare for a few weeks or months before then subsiding for a while [2].

**Figure 1:**
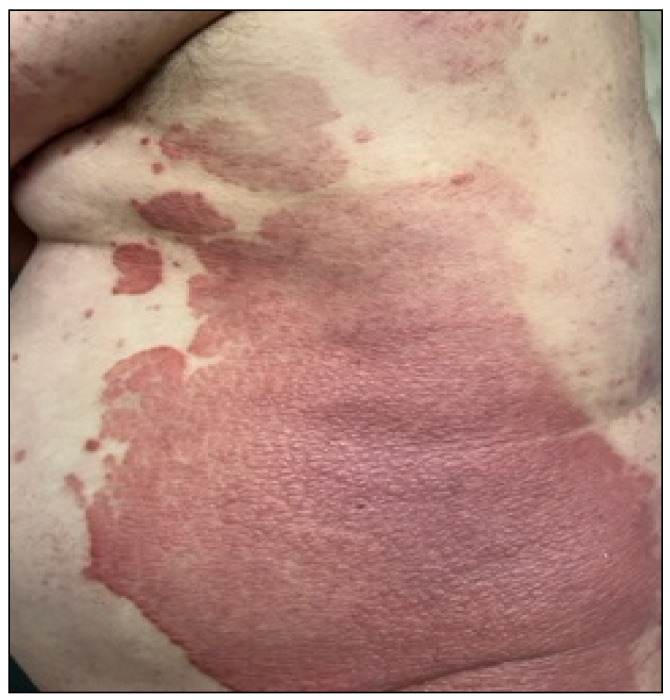
Clinical picture of plaque psoriasis.

The Figure 1 shows symptoms of psoriasis including patchy rashes, scaling, and dry and cracked skin that may bleed. Patients can also suffer from itching, burning, and soreness. There are several types of psoriasis, such as plaque, nail, guttate, inverse, pustular, and erythrodermic psoriasis. Psoriasis is characterized by abnormal differentiation in the cells of the epidermis and hyperproliferation, vasodilation, and inflammatory cell infiltration [2]. Myśliwiec et al. [4] reported that it is well known that psoriasis is linked with metabolic syndrome, obesity, insulin resistance, or cardiovascular disease. Patients with involved joints and severe psoriasis may have a higher risk of mortality [4]. Checa et al. [5] and Jeon et al. [6] described the altered levels of sphingolipids and sphingosine 1-phosphate that were observed in patients suffering from psoriasis [5,6].

## 2. Materials and Methods

This systematic review was written following the 2020 updated Preferred Reporting Items for Systematic Reviews and Meta-analyses (PRISMA) guidelines [7,8]. The review protocol was submitted to the Protocols database (registration number 69618). A medical literature search of PubMed (1996-present), conducted in the winter and spring of 2022, was performed using appropriate terms without date limitations. The main subject of the research was to identify the role of sphingolipid metabolism in the pathogenesis of psoriasis and its comorbidities: cardiovascular diseases, liver disease, and metabolic disorders. Medical subject headline terms included: “sphingolipid’s metabolism in psoriasis”, “sphingolipid’s metabolism in heart disease”, “lipids in the pathogenesis of psoriasis”, “lipidomics psoriasis”, “lipid signaling in psoriasis”, “lipid mediator in psoriasis”, “psoriasis and ceramides”, “psoriasis and S1P”, “psoriasis and sphingosine-1-phosphate”, and “psoriasis and sphingomyelin”. Non-English publications, duplicated publications, and articles with low clinical significance were excluded from the analysis. Original animal and human studies were included in the systematic review. The results of search strings were merged together and duplicates were removed. Afterwards, the titles and abstracts of the remaining studies were screened in order to identify relevant articles that addressed the review subject. Afterwards, the titles and abstracts of the remaining studies were independently screened by two reviewers (M.M. and H.M.) in order to identify relevant articles that addressed the review subject. Disagreements between reviewers were resolved by the opinion of a third reviewer (A.C.). Finally, the selected eligible articles were fully reviewed.

## 3. Results and Discussion

**Figure 2:**
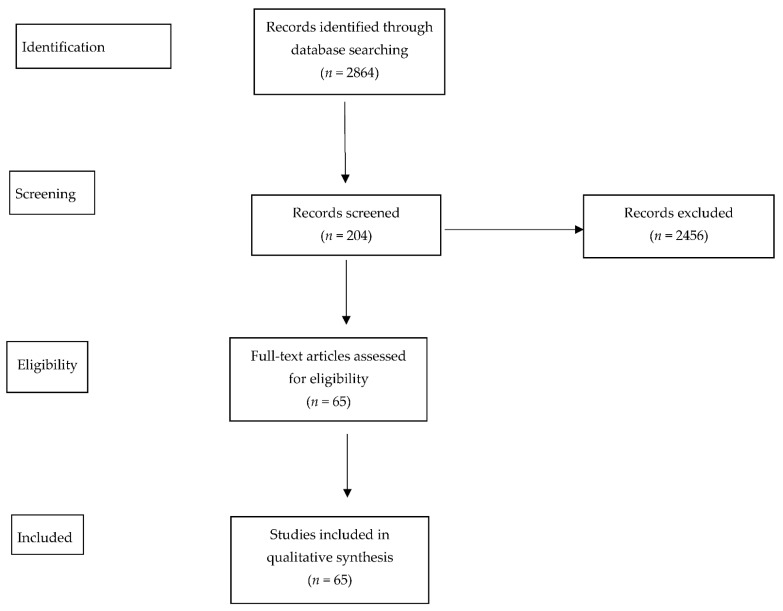
The search process.

The search resulted in the retrieval of 2864 records, of which 204 were screened for relevance and 65 ultimately included in qualitative synthesis, according to Figure 2.

### 3.1. Ceramides—An Overview

Lew et al. [9] highlighted from other studies that ceramides have apoptotic and antiproliferative effects. In psoriasis, the level of ceramides in the epidermis is decreased along with protein kinase C alpha (PKC-α) and c-Jun N-terminal kinase (JNK). PKC-α and JNK are apoptotic cell-signaling molecules that are induced by ceramides. According to the above authors, the loss of ceramides results in downregulation of PKC-α and JNK, which decreases sphingomyelinase-induced ceramide generation and boosts the proliferative and anti-apoptotic features of psoriatic epidermises [9]. Yokose et al. [10] reported that it has been demonstrated that the level of on-hydroxy fatty acid/phytosphingosine base ceramide Cer [NP] and the Cer [NP]/[NS] ratio are higher in the stratum corneum (SC) compared to keratinocytes (KCs) in normal human skin; Cer [NP] is the most abundant ceramide subclass in the SC, while non-hydroxy fatty acid/sphingosine base ceramide (Cer [NS]) is the most abundant Cer subclass in KCs [10]. Lee et al. [11] additionally reported that the genes involved in sphingolipid metabolism had been proven previously (e.g., ceramide glucosyltransferase (UGCG, also known as glucosylceramide synthase), acid beta-glucosidase (GBA), and acid sphingomyelinase (SMPD1)); UGCG transfers glucose to ceramide to form glucosylceramide, GBA plays a role in hydrolyzing glucosylceramide, and SMPD1 transfers sphingomyelin to ceramide. Moreover, during keratinocyte differentiation, glucosylceramide is synthesized by the UGCG enzyme, and glucosylceramide is stored in intracellular lamellar granules and then released into the intercellular space where it is hydrolyzed by GBA to ceramide, the major lipid component of the epidermal barrier [11]. Alessandrini et al. [12] reported on decreased levels of glucosylceramide-b-glucosidase (GlcCer’ase) that were observed in the skin of patents with psoriasis vulgaris compared to normal controls. In addition, lesional psoriatic skin showed an increased level of GlcCer’ase compared to non-lesional skin in all cases, both at the RNA and at the protein level [12].

### 3.2. Sphingosine 1-Phosphate (S1P) Involvement in Psoriasis Pathogenesis

Jeon et al. [6] found that S1P is a bioactive substance that regulates cell growth and differentiation. S1P is an essential regulator of the differentiation and proliferation of human keratinocytes [6]. Moskot et al. [13] suggested that S1P is responsible for angiogenesis, IL-17 uprising, Th17 cell development, and T cell migration [13]. Kozłowska et al. [2] noted that S1P is an insulin resistance and obesity marker. The concentration of S1P in plasma is correlated with metabolic syndrome in obesity. Patients suffering from psoriasis have higher levels of S1P than healthy people. It has been demonstrated that the amount of S1P in psoriatic patients differs depending on the patient’s weight [2]. Sorokin et al. [14] discovered that decreased S1P levels may be linked with the keratinocyte hyperproliferation observed in psoriasis. Oxidized low-density lipoprotein (LDL) has been shown to be stored in psoriatic lesional skin, along with increased levels of low-density lipoprotein receptor-related protein 1 (Lrp1) and the apoB protein. The increased lipolysis and β-oxidation noted in patients suffering from psoriasis might be explained by higher skin tissue demand in bioactive lipid mediators that are involved in revealing inflammation processes [14]. Ji et al. [15] found that sphingosine-1-phosphate receptor 1 (S1PR1) modulators could mitigate psoriatic symptoms in animal models. A selective S1P1 receptor agonist (Syl930), which selectively modulates S1PR1, weakened the thickening of back skin in a sodium lauryl sulphate-induced mouse model of psoriasis and induced other strong antiproliferative and anti-inflammatory effects, such as the alleviation of acanthosis and parakeratosis and the thickening of the epidermis, in a propranolol-induced guinea pig psoriasis model when administered orally [15]. Hong et al. [16] showed the influence of a novel SphK1 activator, K6PC-5, on Ca^2+^ signaling in human keratinocytes, as well as on epidermal proliferation and differentiation in both murine skin and cultured cells. The activation of SphK1 by K6PC-5 was revealed while new substances derived from bioactive and short-chain pseudoceramides were being developed to promote keratinocyte differentiation; K6PC-5 represents a compound with novel activities not anticipated from its simple short-chain pseudoceramide structure. Interestingly, these results reveal that K6PC-5 and S1P have an antiproliferative effect on the epidermis of murine skin [16]. Schuster et al. [17] suggested that attenuated chemokine (C-C motif) ligand 2 (CCL2) production translates to a lowered influx of Mφs (macrophages) into inflamed tissue. Mφs and monocytes take part in auto-immune diseases such as psoriatic arthritis or MS (multiple sclerosis). Elevated CCL2 production in resident Mφs after activation of S1pr4 (sphingosine-1-phosphate receptor 4) suggests that S1pr4 acts as an enhancer of the initial inflammatory response, which fits the observation that S1P levels are often elevated in inflammatory diseases [17]. Setyawan et al. [18] proved that treatment with sphingosine 1-phosphate (S1P) receptor modulators seemed to be associated with decreased rates of thromboembolic events (TEs) and had a protective effect on TEs; however, significant proof was only found for ischemic stroke (IS) [18]. Rujimongkon et al. [19] demonstrated that sericin reduced sphingosine-1-phosphate lyase (SPL) expression in psoriatic skin. This result suggests that the expression of SPL in response to sericin-treated psoriatic skin decreased immune response and keratinocyte proliferation. SPL plays an enzymatic role and is active in immune responses and autoimmune diseases. SPL deficiency impaired neutrophil trafficking into inflammatory tissues. Decreasing SPL expression reduces cell proliferation and induces keratinocyte differentiation. This proof suggests that SPL is mainly active in the immune response process and functions in the regulation of cell development [19]. Okura et al. [20] found that fingolimod is similar in chemical structure to S1P and behaves as an antagonist of subtypes of S1P receptors, for instance S1P receptors 1, 3, 4, and 5, after phosphorylation by sphingosine kinase in vivo. It has been shown that application of fingolimod ameliorated imiquimod (IMQ)-induced psoriatic dermatitis histologically and clinically. After 6 days, the mRNA expression level of IL-17A was decreased in the skin of fingolimod-treated mice. Flow cytometric analyses revealed that fingolimod reduced the percentage and the number of IL-17 producing T cells infiltrating into the skin, whereas it increased them in inguinal lymph node-induced psoriasis by hindering IL-17A-producing T cells from emigrating from the inguinal lymph nodes to the skin [20]. Interestingly, Shin et al. [21] proved that HWG-35D functions as a highly selective sphingosine kinase 2 (SK2) inhibitor and blocks ex vivo Th17-development. Viable use at concentrations as low as 100 nM suggests that the development and optimization of SK2 inhibitors with drug-like properties for treatment of psoriasis is warranted. However, erythema was not eradicated with half dosages of HWG-35D, though thickness and scaling were significantly reduced. In that study, SK2 inhibition using HWG-35D reduced suppressor of cytokine signaling (SOCS1) levels during Th17 polarization. SOCS1 is critical in regulating Th17 differentiation by maintaining signal transducer and activator of transcription 3 (STAT3) and small mothers against decapentaplegic (Smad) transcriptional activities. Th17 differentiation is markedly reduced in SOCS1-deficient naïve CD4^+^ T cells or in suppressor of cytokine signaling 3 (SOCS3) overexpressed T cells [21]. Bocheńska et al. [22] demonstrated in their study that elevated SPHK1 gene activity in psoriatic lesioned skin can generate excessive synthesis of sphingosine phosphate 1, which results in the production of tumor necrosis factor alpha (TNF-α). Consequently, it may activate the nuclear factor kappa light chain enhancer of activated B cells (NF-κB) pathway or secretion of interleukin 8 (IL-8) [22].

### 3.3. Phospholipids in the Pathogenesis of Psoriasis

Shao et al. [23] showed that the fusion of TNF-α and IL-17A is a strong booster of phospholipase A2 Group IIF (PLA2G2F), phospholipase A2 group IVD (PLA2G4D), and phospholipase A2 group IVE (PLA2G4E) expression. Lipidomic analyses showed that phospholipase A2 (PLA2) impacts the mobilization of a phospholipid–eicosanoid pool, which is altered in psoriatic lesions and functions to boost immune responses in keratinocytes. Moreover, overexpression of PLA2G2F (one of the sPLA2s) in a transgenic mouse model led to the progress of chronic epidermal hyperplasia and hyperkeratosis, similar to what has been observed in the pathology of psoriasis. PLA2G4D, which is a cPLA2, is raised in psoriatic mast cells and facilitates CD1a expression, which can be identified by lipid-specific CD1a-reactive T cells that result in the production of IL-22 and IL-17A. Therefore, lipid metabolism and PLA2 enzymes have a pathogenic role in psoriasis. In psoriasis and pityriasis rubra pilaris (PRP) skin, the three PLA2s are mainly visualized in the epidermis, with small effect on other cell types. Of the three upregulated PLA2s where this was explored, PLA2G2F was identified in skin homeostasis and is expressed in the suprabasal epidermis, where it is upregulated during calcium-induced differentiation or stimulation with IL-22. It is involved in hyperproliferative epithelial diseases such as psoriasis and skin cancers. PLA2G4D, a cPLA2, was demonstrated to be expressed in psoriatic mast cells, prompt the release of exosomes, and contribute to the generation of neolipid skin antigens present in CD1a-reactive T cells, which contributes to the output of IL-22 and IL-17A. In addition, resolvin D1, a docosahexaenoic acid (DHA)-derived anti-inflammatory eicosanoid, is lowered in psoriatic skin and downregulated by PLA2s, which ensures a protective role in psoriasis-like dermatitis [23]. Li et al. [24] demonstrated that, in different studies, the levels of leukotriene D4 and leukotriene E3 in serum were significantly higher in psoriasis patients than in healthy controls. The general content of chenodeoxycholic acid in psoriasis vulgaris was reduced compared to healthy controls. It is assumed that the scarcity of bile acid might be involved in the pathogenesis of psoriasis [24]. Farwanah et al. [25] note that the ceramide amount in the uninvolved skin of atopic dermatitis (AD) and psoriasis patients is reduced when compared to healthy skin, with the reduction being more emphasized in psoriasis patients [25]. Moon et al. [26] demonstrated that sphingosine and sphinganine amounts are significantly elevated in psoriatic epidermis compared to non-lesional epidermis and that boosted expression of ceramides is positively linked with the clinical severity of psoriasis [26]. Interestingly, Łuczaj et al. [27] demonstrated that the levels of several species of different groups of phospholipids, including phosphatidylcholines (PC), phosphatidylinositols (PI), phosphatidylserines (PS), and ether-linked phosphoethanolamine (PEo), are decreased in the keratinocytes of patients suffering from psoriasis. The decrease in PC content may be due to the increased activity of lecithin-cholesterol acyltransferase (LCAT), which carries fatty acids from PC to cholesterol. Different possible mechanisms leading to a reduction in PC levels may be connected with the movement of acyl chains from PC to SM that is catalyzed by PC-SM transacylase, which is an enzyme also occurring in keratinocytes. The process of phospholipid transformation catalyzed by cyclooxygenase and lipoxygenase results in the generation of D-series prostaglandins and J-series prostaglandins. These mediators, through various metabolic pathways, boost apoptosis of keratinocytes. Moreover, reactive oxygen species (ROS)-dependent lipid peroxidation end creates, namely 4-hydroxy-2-onenal (4-HNE) and 4-hydroy hexenal (4-HHE), which improve apoptosis through their commitment in the receptor pathway of apoptosis. Increased ROS production and reduced antioxidant capacity results in higher lipid peroxidation. Consequently, this increase may explain the reduction in PS and PI levels noted in keratinocytes of patients with psoriasis. In that study it was also suggested that the scarcity of PI species could be the result of their increased phosphorylation to phosphoinositides by phosphatidylinositol kinases with elevated activity in the psoriatic epidermis. Furthermore, that analysis shows that ultraviolet B (UVB) irradiation causes upregulation of PC species, PC plasmalogens (PCp), and PEo species, all of which are downregulated in non-irradiated psoriatic keratinocytes. One possible explanation for this may be a significant rise in lipid peroxidation in the keratinocytes of patients suffering from psoriasis, which was recently reported. Treatment of keratinocytes from psoriatic patients with cannabidiols (CBD) leads to successive significant reductions in the levels of PC, PS, and most PEo species in the continuation of the pro-apoptotic changes observed in these cells during the development of psoriasis. These changes are compliant with the increased oxidative stress and inflammation process in psoriatic keratinocytes treated with CBD. Regardless of the general tendency towards a reduction in phospholipid levels, the content of PEo molecular species, namely PEo (36:1) and PEo (40:4), is considerably elevated in psoriatic keratinocytes treated with CBD. This finding indicates that treatment with CBD partially prevents the upregulation of PC, PCp, and PEo, as seen in keratinocytes of healthy individuals exposed to UVB radiation. However, treatment of UVB-irradiated psoriatic keratinocytes with CBD results in a significant decline in the level of SM. In contrast, the increase in SM observed in psoriatic keratinocytes not exposed to UVB radiation may suggest the potential role of CBD in increasing epidermal water loss. This lack of water can lead to cell death, which is characteristic for psoriatic keratinocytes. Therefore, it is suggested that the discovered response of keratinocytes in psoriasis patients to CBD treatment is more helpful when performed in the absence of UVB phototherapy. Nevertheless, results of the latest studies on psoriatic patients have proven that topical treatment with an ointment enriched in CBD greatly improves skin parameters, symptoms, and the PASI. Psoriatic keratinocytes represent an increase (in negative zeta potential) compared to control keratinocytes, probably due to changes in the composition of the membrane of phospholipids. Negative zeta potential indicates translocation of PS to the outer layer of the membrane. CBD treatment significantly reduced the negative zeta potential. This result was only noted in keratinocytes from patients with psoriasis. With UVB irradiation, the negative zeta potential skyrocketed remarkably in comparison to the control keratinocytes. Control keratinocytes treated with CBD after UVB irradiation were characterized by substantially reduced negative zeta potential (by up to 27%) compared to irradiated keratinocytes. Irradiation with UVB caused a decline in the negative zeta potential of psoriatic keratinocytes (by 25%) compared to the psoriatic group. In contrast, treatment with CBD after UVB irradiation did not result in a statistically significant alteration in negative zeta potential, which contrasts with psoriatic keratinocytes treated with CBD after UVB exposure [27]. Zeng et al. [28] found that lysoglycerophospholipids (for example as lysophosphatidic acid (LPA) and lysophosphatidylcholine (LPC)) and glycerophospholipid metabolism (including phosphatidic acid (PA), phosphatidylcholine (PC), and phosphatidylinositol (PI)) were substantially heightened in plasma from patients with psoriasis. LPC and LPA are the most prominent lysoglycerophospholipids and are considered to be inflammatory lipids, which take part in several immune-mediated diseases such as atherosclerosis and the autoimmune disease systemic lupus erythematosus (SLE). In that study, the authors found that LPC was significantly heightened in psoriasis plasma and that the opposite was seen for PC [28]. Kim et al. [29] provided evidence that lysophosphatidic acid receptor 5 (LPAR5) takes part in NOD-like receptor family pyrin domain containing 3 (NLRP3) inflammasome activation in macrophages of psoriatic lesions and in keratinocyte activation to output inflammatory cytokines, which results in psoriasis pathogenesis. During tests on the effects of ki16425, an LPAR1/3 antagonist, on IMQ-induced psoriasis-like mice, ki16425 was shown to mitigate IMQ-induced psoriasis-like symptoms, mostly by inhibiting keratinocyte hyperproliferation. The authors also found that ki16425 significantly inhibited mRNA expression of inflammatory cytokines such as TNF-α, IL-6, IL-17, and IL-36γ in skin lesions, as well as PASI levels. IL-36γ, an IL-1 family cytokine, is secreted from keratinocytes stimulated by TNF-α and IL-17 in the psoriatic epidermis to enable T helper subset polarization and promote self-amplifying loops. LPA boosted Rho-associated protein kinase 2 (ROCK2) and PI3K/AKT signaling pathway-mediated cell cycle transformation and proliferation in keratinocytes. Ki16425 or LPAR1 knockdown inhibited these processes and stopped cell proliferation, hence leading to lower IMQ-induced psoriasis-like symptoms [29]. Pang et al. [30] found that, in a Chinese community, the fasting serum values for total cholesterol (TC), high-density lipoprotein cholesterol (HDL-C), low-density lipoprotein cholesterol (LDL-C), and apolipoprotein A-I (ApoA-I) in a patient group with vulgaris psoriasis were all significantly reduced compared to those in healthy controls. The levels of fasting serum triglycerides (TG) and apolipoprotein B100 (ApoB_100_) did not show any significant discrepancies between the patient group and healthy controls. In that study, 86 patients with psoriasis were divided into three groups based on PASI score: mild (PASI < 12, *n* = 11), moderate (PASI ≥ 12 and <30, *n* = 54), and severe (PASI ≥ 30, *n* = 21) groups. In the first group, the levels of ApoA-I and HDL-C were decreased compared to the healthy control group. In the moderate and the severe group, the values for TC, LDL-C, HDL-C, and ApoA-I were all lower than the healthy control group. The levels of TG and ApoB_100_ remained unchanged with disease status when compared to the healthy control group. Interestingly, comparing the different groups of psoriasis patients, the levels of HDL-C and ApoA-I varied substantially. Specifically, the values for HDL-C and ApoA-I were remarkably lower in the severe group compared to the moderate group [30]. Ferretti et al. [31] noted higher levels of lipoprotein(a) (Lp(a)) in the serum of patients with psoriasis compared to control group. Elevated levels of lipid hydroperoxides and lower paraoxonase 1 (PON1) activity were observed in the serum of patients in contrast to healthy subjects, confirming that psoriasis is associated with oxidative stress. The imbalance between oxidative stress and antioxidant enzymes and the elevation of Lp(a) serum levels were related to the extent and severity of psoriasis. Finally, outcomes demonstrated that Lp(a) levels were positively correlated with markers of lipid peroxidation and negatively related to PON1 activity, implying that subjects with higher levels of Lp(a) are more exposed to oxidative damage [31]. Tyrrell et al. [32] revealed that zic family member 1 (ZIC1) was marked with a probable main regulator of many genes that are present in the oxidized ceramide pathway. This is made of several enzymes that directly oxygenate ceramides *ALOX12B*, *ALOXE3*, and *SDR9C7*, as well as downstream genes such as *TGM1*, which encodes the transglutaminase that couples hydrolyzed omega hydroxyl ceramide to involucrin (*IVL*) and other corneocyte lipid envelope (CLE) proteins. Some genes are upregulated in psoriasis, for instance, antimicrobial proteins, *SERPINs*, and *S100s.* Another zinc finger protein, ZNF450, manages to act as a keratinocyte proliferation regulator, and mutations in ZNF450 were implicated in the development of psoriasis [32]. Yamamoto et al. [33] demonstrated that, in psoriasis and skin cancer, the secreted phospholipase (sPLA_2_-IIF) is upregulated in the thickened epidermis and prompts epidermal hyperplasia through production of the unique lysophospholipid plasmalogen lysophosphatidylethanolamine (P-LPE). Some alterations in *Pla2g2e*^−/−^ skin under normal conditions were enhanced to examine the impact of *Pla2g2e* scarcity on these skin disorders. However, neither imiquimod (IMQ)-induced psoriasis nor dinitrofluorobenzene (DNFB)-induced contact dermatitis were agitated in *Pla2g2e*^−/−^ mice in comparison with *Pla2g2e*^+/+^ mice. This was in contrast to *Pla2g2f*^−/−^ mice, where ear swelling was significantly ameliorated in both models. Moreover, although the level of P-LPE, a main metabolite produced by sPLA_2_-IIF, was selectively reduced in IMQ-treated *Pla2g2f*^−/−^ skin relative to WT mice, as noted previously, the levels of P-LPE and other lipid metabolites were similar to the psoriatic skins of *Pla2g2e*^−/−^ and WT mice. Unlike sPLA_2_-IIF that promotes epidermal hyperplasia, sPLA_2_-IIE plays a minimal role in these skin disorders, further emphasizing the functional segregation of these two sPLA_2_s in the skin. Furthermore, *Pla2g2f*^−/−^ mice display attenuated psoriasis with a concomitant reduction in the lysophospholipid P-LPE, whereas skin edema and lipid profiles in these disease models are barely affected in *Pla2g2e*^−/−^ mice. These differences can be clarified, at least in part, by explicit localizations of these two sPLA_2_s in skin niches as well as by their distinct substrate specificities, which could have different impacts on epidermal homeostasis and diseases. This belief also contrasts with the worsening of psoriasis and contact dermatitis with lifted Th1/Th17 immune responses in mice missing sPLA_2_-IID, a “resolving sPLA_2_” that is expressed in dendritic cells and which regulates the functions of immune cells rather than keratinocytes by producing ω3 polyunsaturated fatty acids (PUFA)-derived pro-resolving lipid mediators. In contrast to sPLA_2_-IIF, which selectively gathers P-LPE in psoriatic skin, sPLA_2_-IIE appears to mobilize different unsaturated fatty acids and lysophosphatidylethanolamines (LPEs) in normal skin. Consistent with these in vivo data, sPLA_2_-IIE unleashes these fatty acids and LPEs in an in vitro enzyme label using a skin-extracted phospholipid mixture as a substrate. Given its spatiotemporal localization, it is tempting to speculate that sPLA_2_-IIE conveys unsaturated fatty acids and LPEs in hair follicles during anagen. It has been reported that several PUFA metabolites (e.g., prostaglandins) or LPA variably affect hair growth, quality, and cycling. However, the skin levels of PUFA metabolites and LPA are not profoundly impacted by *Pla2g2e* deficiency, indicating that sPLA_2_-IIE-derived PUFAs are largely uncoupled from downstream lipid mediators [33]. Gaire et al. [34] reported that suppressing LPA_5_ activity with a pharmacological antagonist alleviated imiquimod (IMQ-induced) psoriasis-like symptoms. It also attenuated macrophage infiltration into psoriasis lesions. Activation of LPA_5_ signaling was found to upregulate macrophage NLRP3 expression in psoriasis lesions. Interestingly, in vitro studies revealed that LPA could activate NLRP3 inflammasome in lipopolysaccharide (LPS)-primed macrophages through LPA_5_ [34]. Syed et al. [35] described that, in psoriatic skin, a positive combination of sphingosine kinases (SPHKs) and NLRP3 inflammasome components was noted, with some of these representing targets of NF-κB signaling. Other NF-κB target genes featured in mosaic images, both in healthy and psoriatic skin, pointing out that regulation of NLRP3 inflammasome components might involve other signaling pathways than NF-κB alone [35]. Ogawa et al. [36] found decreased expression of the differentiation-specific proteins keratin 1, involucrin, and loricrin in epidermal fatty acid-binding protein (E-FABP)/keratinocytes relative to E-FABP/keratinocytes. E-FABP expression was elevated in response to increased lipid traffic, which is related to abnormal keratinocyte proliferation and differentiation in psoriasis. It was proven that E-FABP enhances keratinocyte differentiation by boosting the transcriptional activity of peroxisome proliferator-activated receptors (PPARs) by transporting fatty acids directly to PPARs. Altered fatty acid metabolism and abnormal keratinocyte differencing in psoriatic skin may be related to this drastic upregulation of E-FABP expression in psoriasis. Many saturated fatty acids are less abundant in E-FABP/keratinocytes, which is consistent with a previous report showing that E-FABP preferentially binds to saturated fatty acids in vitro. This is a remarkable correlation between E-FABP and fatty acids and the increased expression of E-FABP in psoriasis, and the pathogenesis of psoriasis may be influenced by E-FABP activity through the impaired metabolism of fatty acids and their derivatives [36]. Shou et al. [37] noted the pathogenic role of ferroptosis in sensitizing Th22/Th17-type cytokines, thereby providing candidate markers of psoriasis aggravation. Ferroptosis is a non-apoptotic form of programmed cell death, which is associated with the exertion of damage-associated molecular patterns (DAMPs) and alarmins. The paradigm of ferroptosis comprises lipid peroxidation accumulation and iron overload. It was suggested that the induction of ferroptosis, especially lipid peroxidation of psoriatic keratinocytes, leads to inflammatory responses, which could be rescued by ferroptosis inhibitor Fer-1. Notably, inhibition of ferroptosis with ferrostatin-1 (Fer-1) is beneficial for the treatment of psoriasis. Psoriasis is connected with irregularities in lipid metabolism, free radical generation, and lymphokine production. The levels of nitric oxide, ROS, malondialdehyde (MDA), and epidermal iron in psoriasis patients were increased, while at the same time the levels of superoxide dismutase, glutathione peroxidase activity, glutathione peroxidase 4 (GPX4) expression, and total antioxidant capacity were decreased. These levels correlated with the severity of the disease. Interestingly, in that study, a significant elevation in Acyl-CoA Synthetase Long Chain Family Member 4 (ACSL4) and 4-HNE-modified protein levels was observed, as well as a reduction in GPX4 in psoriatic lesions, which are crucial regulators in the process of ferroptosis [37].

### 3.4. Sphingolipids in the Pathogenesis of Psoriasis

Moskot et al. [13] stated that an epidermal barrier in psoriasis is known for amended sphingolipid levels. In the composition of ceramides in psoriasis, very long chain (VLC) ceramides and non-hydroxy fatty acid/sphingosine base ceramide CER[NS] are increased, and ultra-long chain (ULC) ceramides are decreased. In psoriasis, a common phenomenon is trans epidermal water loss (TEWL) and lower retention capacity, which is due to lower levels of phytosphingosine-carrying ceramides, for instance on-hydroxy fatty acid/phytosphingosine base ceramide Cer[NP], alpha-hydroxy fatty acid/phytosphingosine base ceramide Cer[AP], and acylceramides (acylCer). Decreased acylCer reflects hyperproliferation in psoriasis and the permeability barrier, and the reduced amount of ceramides may generally be reflected by boosted ceramide degradation [13]. Holleran et al. [38] revealed that, in psoriasis, a decrease in ceramide synthases (CERS), elongases (ELOVLs), very long FAs (longer than C26), ceramides NP, and on-hydroxy fatty acid/6-hydroxy-sphingosine base NH was observed, although it is worth noting that ceramides NS with shorter FAs (shorter than C24) are increased. Those changes all correlated with the abnormal function of the epidermis. It is also proven that ceramide synthase 3 (CERS3) and ELOVL4 are decreased in interferon gamma (IFN-γ) stimulated keratinocytes. Nevertheless, IFN-γ is increased in psoriatic skin and may be related to epidermal dysfunction. These changes may lead to inflammation, epidermal hyperplasia, and, finally, to the exacerbation of psoriasis. Looking at the inflammatory process in psoriatic skin, it is worth mentioning that levels of IL-6 and IL-8 are increased, both of which are typical psoriasis-associated proinflammatory cytokines. This rise is caused by psoriasis-like HPKs (Human Primary Keratinocytes), and even the psoriasis marker DEFB4A was also observed to have increased. That definitely shows that keratinocytes from psoriatic lesions are very similar in their function to psoriasis-like HPKs. In psoriatic non-lesional skin, decreased mRNA expression of glucosylceramide-β-glucosidase is observed compared to healthy skin. Interestingly, the mRNA level of this enzyme was higher in psoriatic plaques than in non-lesional skin. Psoriatic lesions (both active-type and chronic-type plaques) were characterized by a reduction in prosaposin in comparison to psoriatic non-lesional skin and healthy skin. Prosaposin, an essential forerunner of saposin and a nonenzymatic cofactor, is required in the process of sphingolipid hydrolysis by, for example, β-glucocerebrosidase. The enzymatic transformation of glucosylceramides to ceramides may be disturbed by reduced levels of prosaposin and saposin in psoriatic skin [38]. According to Wang et al. [39], PsA (psoriasis arthritis) patients had significantly higher levels of ApoB, TC, and LDL and a higher ApoB/ApoA1 ratio than psoriasis without arthritis (PsO) patients [39]. Myśliwiec et al. [40] observed lower concentrations of fatty acids in psoriatic patients and in the PsA group. It was noted that ample amounts of interferon-γ were present in psoriatic skin lesions. This decreases the mRNA expression of elongases of long-chain fatty acids (FA), which is involved in FA metabolism and chain elongation. This could have led to the observed FA profile in that analysis. Higher percentages of saturated fatty acids (SFA) and subdivided into monounsaturated fatty acids (MUFA) and a lower percentage of polyunsaturated FA (PUFA) in both psoriatic groups compared to the healthy controls were also observed. It is widely known that SFA have proinflammatory effects. In contrast, MUFA are believed to have an anti-inflammatory function. Eicosanoids, which are derived from n-6 PUFA arachidonic acid (prostaglandins and leukotrienes), can worsen inflammatory processes, and oxylipins generated from n-3 PUFA (resolvins, maresins, protectins) characterize anti-inflammatory features. The noticed rise in SFA percentage in the PsA group may show a potential connection between PsA and the increased risk of metabolic comorbidity, as SFA are generally linked to higher cardiometabolic risk. The significant distinction in FA profile between psoriasis and PsA in that study was the higher SFA/UFA ratio in the PsA group. It was proven that SFA/UFA ratio is higher in obese psoriatic patients and in individuals with hypertension. Moreover, SFA/UFA ratio correlated positively with disease duration. The authors suggested that the higher SFA/UFA ratio in psoriatic arthritis may indicate a cardiometabolic risk profile in psoriasis and in psoriatic arthritis [40]. Souto-Carneiro et al. [41] found that lipid β-methylenes, lipid α-methylenes, and lipid polyunsaturated allylic methylenes express substantially differently in PsA and seronegative rheumatoid arthritis (negRA) patients, with all methylenes higher in PsA patients. Lipid β-methylenes and lipid α-methylenes can mostly be associated with changes in the levels of lipids in the serum. Since they reflect the lipid β-methylenes and α-methylenes common to most medium and long-chain fatty acids, the lipid polyunsaturated allylic methylenes reflect polyunsaturated allylic methylenes because of the presence of PUFAs, which are known to play a central role in the homeostasis of the immune system. PUFAs have been associated with both proinflammatory (ω6-PUFAs) and anti-inflammatory (ω3-PUFAs) capabilities [41]. Gao et al. [42] reported that, in the early stages of psoriasis, PLA2G4B expression rose due to stress response, which caused abnormal lipid metabolism. The release of free fatty acids and lysophospholipids can be presented to CD1b through activation of myeloid dendritic cells. This causes naïve CD8þ T cells and Th17 to produce inflammatory factors such as IL-17 and IL-36. The increase in the local concentration of inflammatory factors is involved in lipid metabolism and immunological dysregulation response, further aggravating inflammation of the skin lesion site and leading to excessive proliferation of keratinocytes. After using an siRNA emulsifier (siRNA505 PLA2G4B liposome emulsifier and betamethasone), the fluorescence intensity of PLA2G4B decreased significantly, which means that the emulsifier can penetrate the skin surface to play a key role in that process [42]. Tawada et al. [43] demonstrated that IFN-γ downregulates elongation of very long chain fatty acids protein 1 (ELOVL1) and CERS3, which in turn lowers the levels of CERs with long-chain fatty acids in human skin. Additionally, the authors showed that the proportion of CER[NH] with a total carbon number between 40 and 43 (C40–C43 CER[NH]) was elevated, whereas that of C47–C50 CER[NH] was decreased in AD and psoriasis patients compared to healthy controls. The proportion of C44–C46 CER[NH] was decreased only in psoriasis patients. Similar changes in CER[ADS]/[NP] and CER[AP] levels were observed in psoriasis but not in AD; the proportion of C38–C43 CER[ADS]/[NP] and C38–C42 CER[AP] was increased, whereas that of C49–C52 CER[ADS]/[NP] and C45–C48 CER[AP] was decreased. A significant difference in the proportion of C44–C48 CER[ADS]/[NP] was also observed between psoriasis patients and controls. No obvious difference was observed in the proportion profiles of CER[AH]/[NS] and CER[NDS]/[AS] among psoriasis, AD, and control groups [43]. Additionally, Utsunomiya et al. [44] showed that acylceramide (acylCer) and its derivative protein-bound ceramide have an essential role in skin permeability barrier formation. Modestly reduced levels of some ceramide species, including esterified omega-hydroxyacyl-sphingosine (EOS) which is a representative acylceramide, were consistent with mild skin barrier dysfunction in dermokine (DMKN)-αβγ-/- mice. Additionally, the lipid droplets detected in the stratum corneum may be reflecting the defective secretion of lamellar body content. Furthermore, levels of protein-bound 16 ceramide were also modestly decreased, and this may be associated with the slight fragility of CE in DMKN-αβγ-/- mice. These findings suggest that DMKN isoforms are essential regulators of barrier function and skin inflammation. DMKN-deficient mice may be useful for evaluating disease pathogenesis and preclinical assessment of treatments aimed at specifically targeting molecular drivers of congenital ichthyosis and psoriasis. Furthermore, recombinant DMKN or a therapeutic strategy to enhance DMKN function may be effective for these skin disorders [44]. Del Rosso et al. [45] found that an application of a ceramide/keratolytic-containing cream and/or cleanser provided significant patient-perceived benefits within two weeks in patients dealing with psoriasis. Moisturizers/barrier repair formulations and keratolytic/desmolytic agents are clinically recognized as valuable topical treatment agents for psoriasis. Ceramide-containing products offer beneficial effects in improving barrier function, reducing TEWL, and maintaining SC hydration. In that study, nearly all patients responded positively to the aesthetic attributes of each formulation (i.e., nongreasy, absorbed quickly and easily) and overall product performance (i.e., skin looks and feels healthier). Additionally, a treatment regimen of twice-daily cream application with twice-weekly cleanser use appeared to provide additional benefits based on the perceptions of the study patients. Patients responded positively regarding the use of both products together, with self-reported improvements in their skin (e.g., improved feel, appearance, and symptom relief), even in those who experienced psoriatic flares. Further clinical studies using the ceramide/keratolytic-containing cream and cleanser regimen in combination with topical therapy in patients with psoriasis would provide clearer insight into the importance of barrier repair in patients with psoriasis and the potential benefit of this approach in reducing flares of the disease [45]. Choi et al. [46] and Motta et al. [47] noted that the major change in ceramide composition in psoriasis was associated with a significant drop in the percentage of phytosphingosine-carrying ceramides (3 and 6I) compared to normal stratum corneum, together with increases in some sphingosine-carrying ceramides (2I, 2II, and 5I). Phytosphingosine-carrying ceramides accounted for 25% of total ceramides in the psoriatic scale versus 44% in normal stratum corneum. In another study, the ceramide fractions and transepidermal water loss in psoriatic and normal stratum corneum were compared. In all types of psoriatic scale, ceramide 1, 3, 4, 5II, and 6I content was decreased, and ceramide 2I, 2II, and 5I content was increased. The mean transepidermal water loss value of scaling psoriatic plaques was 11.5 g/cm^2^/h, compared to 4.3 g/cm^2^/h in controls. They also compared the total content of the three main intercellular lipids in psoriatic scales and normal human stratum corneum. The molar ratio of free fatty acid/cholesterol/ceramide in normal human stratum corneum was 4.1:1.3:1 compared with 2.2:1.3:1 in psoriatic scales. The relative free fatty acid content was much lower in psoriatic scales compared to normal human stratum corneum, while ceramide and cholesterol content was slightly increased in psoriatic scales compared to normal human stratum corneum. These investigators also identified a change in sphingomyelinase expression in psoriatic skin stratum corneum. In psoriatic lesional skin, immune localization of sphingomyelinase was definitively lowered in the stratum corneum. Taken together, these findings suggest that, in patients with psoriasis, a decrease in prosaposin and sphingomyelinase might lead to a decrease in ceramide 1 and other ceramides, as has been observed in psoriatic plaques [46,47]. Cho et al. [48] mention that the level of Cer [NP] and the Cer [NP]/[NS] ratio are higher in the Striatum corneum (SC) compared to keratinocytes (KCs) in normal human skin and that Cer [NP] is the most abundant Cer subclass in the SC, while Cer [NS] is the most abundant Cer subclass in KCs. Ceramides bearing the moieties of amide-linked non-hydroxy acid, α-hydroxy acid, or ω-hydroxy acid and ester-linked fatty acids on sphingosine provide the driving force for lamellar assembly, and these structural moieties are thought to be important in maintaining the structural integrity of the epidermal barrier against water loss through the skin. Assessment of the lesional epidermis of psoriasis patients revealed no alteration in the levels of linoleic acid (LA) or other fatty acids compared to the non-lesional epidermis of the same patients. In addition, no relationship was observed between the levels of fatty acids and the PASI score in mild to moderate psoriasis. On the other hand, the level of ceramide synthesis was significantly reduced in the lesional epidermis of patients. These results are in concordance with the results of earlier studies in which decreases in the levels of ceramides, specifically ceramide 1, 4, and 5, have been shown to be associated with increased transepidermal water loss in the psoriatic epidermis. Furthermore, a positive correlation between the percentage reduction in ceramide synthesis in the lesional epidermis and clinical severity was demonstrated in that study [48]. Tessema et al. [49] reported that lecithin-based microemulsions (MEs) were observed to have boosted the penetration of oat CERs in vitro and ex vivo. Moreover, the starch-based nanoparticles (NPs) sustained the release of oat CERs. In both in vitro as well as ex vivo observations, oat CERs from ME gel have shown better degrees of penetration compared to the NP gel. The gel formulations, in general, have shown PhytoCERs targeting into the SC. Therefore, they can be used when localizing the CERs in upper epidermal stratums. To sum up, this analysis gave an insight into the skin permeation profile of oat CERs, which is essential for showing their best use in improving the barrier of affected skin [49]. Egger et al. [50] highlight that caveolae and caveolin-1 (Cav1) are known to be involved in endocytosis. They are flask-shaped invaginations of the cell membrane rich in cholesterol and sphingomyelin. Moreover, Cav1 is extremely stable at the cellular membrane, and only some Cav1 rich vesicles actually become internalized. Cav1 can stabilize cellular membranes and slow down membrane invagination, budding, and vesicle internalization. Cav1 participates in inhibiting keratinocyte differentiation and raises intriguing possibilities for targeting Cav1 in cutaneous disorders manifested by abnormal differentiation and proliferation, such as psoriasis and hypertrophic scarring. Interestingly, Cav1 also locates itself on melanocytes and its expression can be induced by UV exposure, where it leads to changes in cell morphology and increased melanin transfer and skin pigmentation as a result of changes in cAMP production. Soon after, evidence began to emerge demonstrating the connection between downregulated Cav1 and excessive epidermal hyperplasia that is classically seen in psoriasis. Histological analyses of psoriatic skin lesions demonstrated lowered expression of Cav1 compared to unaffected skin. Additionally, one study found markedly decreased expression of Cav1 in different types of psoriasis, including psoriasis vulgaris, localized pustular psoriasis (PP), and erythrodermic psoriasis, with psoriasis vulgaris having the most significant downregulation of Cav1 expression compared to the other two types. There was a significant reduction in Cav1 expression in lesional skin compared to non-lesional skin [50]. Geilen et al. [51] revealed that hydrolysis of approximately 25% of total cellular sphingomyelin was observed after 1α,25-dihydroxyvitamin D treatment, and than SM levels came back to control levels. Similar results were obtained regarding primary keratinocytes. The amount of hydrolyzed SM and the time course for SM hydrolysis are in accordance with results obtained in the leukemic cell line (HL-60). TNFα, another well-known inducer of the SM cycle, also activated SM hydrolysis in keratinocytes and immortalized human keratinocyte cell line (HaCaT) cells. We provide evidence that the SM cycle, a signaling pathway originally described in HL-60 cells, is also found in the immortalized human keratinocyte cell line (HaCaT) and in human keratinocytes. In addition to the known inducers of SM hydrolysis, 1α,25-dihydroxyvitamin D and TNF-α both responded to the antiproliferative, synthetic vitamin D analog calcipotriol. Interestingly, acetylsphingosine, which is a short-chain ceramide similar to the naturally occurring breakdown product of SM hydrolysis, mimicked the effects of calcipotriol and l,25-dihydroxyvitamin D on cell proliferation [51]. Zhang et al. [52] reported that glucose transporter 1 (GLUT1) was also essential for proliferation in human keratinocytes in vitro, indicating that psoriatic lesions have bigger requirements for glucose uptake and metabolism, as evidenced by increased *Glut1* expression and PET scans. Confirmation that glycosylated ceramides and UDP-glucose are essential intermediates for epidermal ceramide maturation, lamellar body formation, and stratum corneum development has been shown in analyses of epidermal lipids conducted in previous reports. Interestingly, liquid chromatography–mass spectrometry and liquid chromatography–tandem mass spectrometry (LC-MS/MS) revealed that the epidermis of *K14.Glut1* mice demonstrated normal levels of phospholipids, lower levels of free ceramides and sphingoidbases, and increased levels of sphingomyelins. Moreover, while hexosylceramide levels were much lower in keratinocytes from *K14.Glut1* mice, they were not significantly decreased in the epidermis of *K14.Glut1* neonatal mice compared to WT. Specifically, glycosylated ceramides have been essential for the proper formation of an intact stratum corneum, as the deletion of glucosylceramide synthase (*Ugcg*) results in a disruption of normal ceramide metabolism, aberrant stratum corneum development, and perinatal lethality. Despite defects in hexosylceramide synthesis in vitro, it was proven that the skin barrier was intact, indicating that the epidermis is able to maintain sufficient levels of ceramides for the maturation of epidermal ceramides even in the absence of Glut1 [52]. Li et al. [53], in their results of a multicenter evaluator-blinded randomized controlled trial (RCT), demonstrated the superiority of the linoleic acid-ceramide-containing moisturizer (LA-Cer) in continuous improvement of PASI 50 response after topical glucocorticoid (GC) use over the control group. Interestingly, the topical linoleic acid-ceramide moisturizer (LA-Cer) prevented relapses and adverse events (AEs) after the end of corticotherapy [53]. Liu et al. [54] demonstrated that both PC and PS were lowered in the IMQ-induced groups but upregulated by (*R*)-salbutamol treatment, which indicates that (*R*)-salbutamol conferred anti-psoriatic effects by modulating the metabolism of glycerophospholipids. In previous articles, the importance of sphingolipids in innate immunity regulation was shown, especially in T cell differentiation and programming. In that study, it was discovered that (*R*)-salbutamol acted against IMQ-induced psoriasis by regulating the Th17/Treg axis. The metabolomics results suggest that the regulation of Th17/Tregs by (*R*)-salbutamol may participate in sphingolipid metabolism. The biomarkers which were identified in this article, either in IMQ-induced mouse psoriasis or after (*R*)-salbutamol treatment, could be useful in identifying pathways and mechanisms mediating the pathogenesis of psoriasis [54]. Simoni et al. [55] reported that psoriasis coexists with invariant natural killer T (iNK T) cell activation. Additionally, the disease is characterized by elevated CD1d expression in psoriatic lesions. Interestingly, CD4^+^ iNK T cells infiltrate lesions in psoriasis and might be pathogenic. This suggests that protection or worsening of autoimmune diseases by iNK T cells may be due to disequilibrium between the different subsets. Microbial infections enhance the expression of glucosylceramide synthase, leading to the synthesis of β-glucosylceramide (β-GlcCer). Moreover, this self-glycolipid presented by CD1d activates iNK T cells and promotes their proliferation. iNK T cell function may be activated by boosting the expression of glucosylceramide synthase that increases presentation of self-glycolipids, which are able to activate iNK T cells [55]. Breiden et al. [56] note that the final step of triacylglyceride biosynthesis is the acylation of diacylglyceride by acyl-CoA into diacylglycerol acyltransferase 2 (DGAT 2). This is decreased in human skin affected with psoriasis. DGAT 2 shortages in mice lead to disturbed barrier function with increased transepidermal water loss due to drastically reduced total linoleic acid and acylceramide content [56]. Hong et al. [57] measured the expression of SPT and ceramidase in both psoriatic epidermis and non-lesional epidermis. It was concluded that SPT levels were significantly decreased in psoriatic skin. However, the levels of ceramidase showed no significant difference between psoriatic epidermis and non-lesional epidermis. The relationship between PASI score and decreased levels of SPT was also described to highlight the role of decreased SPT in the severity of psoriasis. The link between the percentage reduction in the ratio of SPT/tubulin in the lesional epidermis and the PASI scores showed a significantly negative correlation. Looking at those results, lowered levels of ceramide in psoriatic skin are responsible for decreased levels of SPT in psoriatic skin lesions, and their levels of reduction are highly related to the clinical severity of psoriasis [57].

### 3.5. Sphingolipids in Psoriasis and Obesity and Metabolic Syndrome

Kozłowska et al. [2] reported that lignoceric ceramide (C24:0) and palmitoleic ceramide (C16:1) were inversely correlated with the severity of psoriasis measured by PASI in overweight patients. Lignoceric ceramide is a key factor in maintaining the right epidermal barrier. The negative correlation between C24:0 and inflammatory markers is well known. Ceramide C24:0 inhibits inflammation and the transformation of insulin resistance and maintains the proper homeostasis of the liver. Nervonic ceramide (C24:1) positively correlates with PAS with normal body weight. C24:1 is an inflammatory marker and possesses a prognostic value in predicting the course of psoriasis. There is a positive connection between C24:1 and CRP in both normal weight and obese patients. Ceramide C24:1 is regarded as a protective and anti-inflammatory substance. C24:1 metabolism disturbances may lead to diabetes complications. There was a noticeable decrease in the level of C24:1 in type 1 diabetes, which increased after insulin treatment. In patients with normal weight, an inverse correlation between eicosapentaenoic ceramide (C20:5) and PASI occurs. The amount of ceramides may be altered under eicosapentaenoic acid (EPA) and DHA supplementation [2]. Kozłowska et al. [58] reported that the highest level of S1P was found among normal weight psoriatic patients in comparison to obese psoriatic patients, with an assumption that higher S1P cell uptake exists in obese patients and may lead to reduced insulin sensitivity. Additionally, they reported that there is a well-known connection between metabolic disturbances and Cer profile. In patients with obesity and comorbidities such as type 2 diabetes mellitus (T2DM), an elevated level of ceramides and its subspecies was observed compared to healthy patients. During gastric bypass, lowered plasma levels of C14:0, C16:0, C20:0, and C24:0 in those patients were also observed. The reduction in C24:0 was linked with increased insulin sensitivity and weight reduction. In young obese adults, a positive link between γ-glutamyl transpeptidase and C24 was observed. The levels of C14:0, C16:0, C22:0, and C24:0 were elevated in prediabetic and diabetic nonhuman primates, and the levels of these ceramides correlate with the homeostasis model assessment of insulin resistance (HOMA-R). In conclusion, C24:0 and C14:0 possess a great influence on creating metabolic disturbances, including for patients suffering from psoriasis [58]. Majumdar et al. [59] report that the level of S1P in plasma may be nstable in the insulin-resistant state. High levels of glucose induce sphingosine kinase (Sphk) in vascular endothelial cells in vitro, which generates S1P that subsequently activates vascular endothelial cell growth. It has been proven that a balance between S1P and sphingolipid metabolites is related to cell death and survival, which may lead to the conclusion that those metabolites function as moderators/regulators of insulin resistance and atherogenesis associated with the chronic inflammatory state of obesity [59].

### 3.6. Sphingolipids in Psoriasis and Liver Diseases

Kozłowska et al. [58] report that C14:0 and C24:0 ceramides are involved in the pathogenesis of liver disease. The level of ceramides correlates directly with alanine aminotransferase (ALT) concentration. Myristic ceramide (C14:0) possesses the strongest correlation, which was observed in relation to ALT level. C14:0 is widely considered as a novel biomarker of hepatosteatosis, independent of obesity. Under those circumstances, the high level of C14:0 recorded in patients suffering from psoriasis may be related to liver disease, and it can inform us about progressing or incoming liver disease. Interestingly, it was observed that total serum Cer concentration in patients with psoriasis did not correlate with the ALT level in serum. The serum concentration of myristic ceramide (C14:0) and sphingosine-1-phosphate was higher in patients with high-level ALT than in healthy controls. The serum concentration of lignoceric ceramide (C24:0) correlates positively with the ALT level. To sum up, patients dealing with psoriasis are susceptible to the evolution and transformation of liver disease. Altering levels of ceramides in patient serum with psoriasis may indicate the triggering liver disease [58].

### 3.7. Sphingolipids in Psoriasis and Heart Disease

Hadas et al. [60] revealed that increased ceramide levels in heart tissues during acute myocardial infarction (MI) were associated with higher cell death rates in the left ventricle (LV) and deteriorated cardiac function. Ceramidase, the enzyme which hydrolyzes pro-apoptotic ceramide, generates sphingosine, which is phosphorylated by Sphk to create the pro-survival molecule S1P. It is believed that acid ceramidase (AC) overexpression can alleviate the negative effects of elevated ceramide and promote cell survival, thereby providing cardioprotection after myocardial infarction. High amounts of ceramides in plasma concentrations is associated with a higher probability of MI recurrence and death. High cellular ceramide levels may trigger programmed cell death. It is widely known that ceramide levels are high in the heart tissues of rodents and humans during acute MI, and that blocking de novo ceramide synthesis in rodents can improve heart function post MI. Moderate concentrations of ceramidase and sphingosine kinase inhibitors were not toxic to nrCMs (neonatal rat cardiomiocytes) under normal culture conditions. However, incubating nrCM cells with a higher or lower concentrations of the two inhibitors together induced cell death. Restraining these enzymes may have caused ceramide and/or sphingosine to rise to toxic levels. A combination of hypoxia and enzyme inhibition may boost the rate of pro-apoptotic sphingolipid accumulation and cell death. Restraining AC activity in mouse hearts led to elevated cardiac cell death 24 h post MI. Those results show that AC activity is an essential cardioprotective element in ischemic heart disease, enhances heart function post MI, and moderately diminishes the infarct area and inflammation levels following myocardial injury. Inhibiting AC after an MI episode significantly decreases survival, suggesting AC’s active role is vital to mouse survival post MI. S1P receptor 2 (S1pr2) or Sphk2 overexpression did not significantly diminish cardiac cell death either in vitro or in vivo. Sphk1 is, thus, effective for preventing apoptotic death but is not redundant in relation to Sphk2 for this function. Sphk1 interacts with TNF receptor-associated factor 2 (TRAF2). and this mutual interaction tends to trigger TRAF2’s anti-apoptotic activity. Our pathway analysis for sphingolipid signal transduction revealed upregulated TRAF2 post MI. It has been recently proven that TRAF2 possesses a cardioprotective role [60]. Poss et al. [61] note that the best-characterized ceramide score is CERT1, though most of the ceramide species contained within CERT1 were predictive of CAD. Cer (18:1/24:0) is a good marker of CAD [61]. Kovilakath et al. [62] highlight that S1P signaling components such as S1PR1, sphingosine 1-phosphate receptor 2 (S1PR2), and sphingolipid transporter 2 (SPNS2) are important for cardiomyocyte development and myocardial precursor migration to the ventral midline of the embryo, where they migrate into the primitive heart tube. Proper concentrations of S1P must be maintained for correct development of cardiac valve precursors. Initial studies, including our own, have shown that inhibition of overall sphingolipid biosynthesis prevented cardiac lipotoxicity, suggesting that merely reducing ceramides in the lipotoxic heart may be a “silver bullet”. A cardiomyocyte-specific serine palmitoyltransferase long-chain base subunit 2 (SPTLC2) null mouse model showed an exacerbated cardiac phenotype. In addition to ceramides, however, alterations in dihydroceramides, ceramide-1-phopshates, sphingomyelins, and glycosphingolipids likely play disparate roles in cardiac pathology. Long-chain ceramides and very long-chain ceramides and sphingomyelins are associated with severe cardiac complications. Ceramide (d18:1/16:0), (d18:1/18:0), and (d18:1/24:1) levels were associated with major adverse CVD events. Ceramide (d18:1/22:0) was not associated. There was an increased plasma concentration of C16:0 and C18:0 ceramides in participants with heart failure with preserved ejection fraction (HFpEF). Levels of ceramides and sphingomyelins with a 16-carbon acyl chain length were directly associated with higher risks of mortality from CVD. In contrast, it was observed that levels of ceramides with a 22- or 24-carbon acyl chain length and SM with a 20-, 22-, or 24-carbon acyl chain were associated with lower risks of CVD mortality. While these biomarkers and diagnostic indicators represent advances in identifying and diagnosing CVD, there exists a distinction between long chain (i.e., C16–C18) and very long chain (i.e., C20–C24) ceramides, which are generated by different ceramide synthase isoforms (CERS1/5/6 vs. CerS2/4, respectively). Generally, a lot of circulating lipids are created in the liver, a detailed study of which demonstrates that circulating ceramides but not sphingomyelins come from the endothelium of blood vessels. Endothelia are functionally abnormal in CVDs, which may lead to the plasma sphingolipid profile reported in heart failure [62]. Altekin et al. [63] mention that chronic skin inflammation in patients suffering from psoriasis may lead to atherosclerosis. Interestingly, it was shown that pulse wave velocity (PWV) and the average and maximum carotid intima media thickness (CIMT) values of psoriasis patients were higher than in the healthy group [63]. Shao et al. [23] demonstrated that the ApoB/ApoA1 ratio is a strong and new risk factor for cardiovascular disease (CVD). Data from Indian patients with acute myocardial infarction (AMI) showed that the ApoB/ApoA1 ratio was a better indicator of CAD risk than other lipid ratios, including the TC/HDL-C and LDL/HDL-C ratios. Furthermore, the ApoB/ApoA1 ratio was associated with femoral artery atherosclerosis [23]. Ilanbey et al. [64] note that chitotriosidase (ChT) activity was found to be higher in patients with hypertension than in those with other comorbidities, such as psoriatic arthritis, diabetes mellitus, or hypothyroidism. The higher activity of ChT in hypertension suggests that macrophages may play a greater role in the pathogenesis of hypertension. ChT is an enzyme excreted by activated macrophages and neutrophils as a response to proinflammatory signals [64]. Lasa et al. [65] found twenty-two studies that assessed bradycardia in 9047 patients exposed to an S1P modulator. Selective S1P1 modulators showed a substantially increased risk of bradycardia. Additionally, seventeen studies assessed the atrioventricular (AV) block in 7662 patients exposed to an S1P modulator (fingolimod, ozanimod, siponimod, ponesimod, amiselimod, etrasimod) [65].

The basic characteristics of the original papers in relation to the researched topic are summarized in Table 1, Table 2, Table 3, Table 4, Table 5, Table 6 and Table 7 below.

## 4. Conclusions

In recent times we have experienced quickly growing interest in the topic of sphingolipids metabolism in human diseases. The discoveries made in recent years have proven the huge role that altered levels of ceramides play in psoriatic skin, which leads to anti-apoptotic and pro-proliferative states, the over-proliferation of keratinocytes, and the development of skin lesions. Patients suffering from psoriasis with higher S1P serum concentrations may be predisposed to the development of metabolic syndrome. Finally, ceramides are measured as markers of recurrence and mortality after myocardial infarction, coronary artery disease, and acute coronary syndrome. Although sphingolipid metabolism in human diseases has been broadly researched, further research studies are still needed to evaluate the effect of individual ceramides on the course of various diseases. These studies may help us discover new prognostic factors and new treatment modalities.

## Figures and Tables

**Table 1:** Summary of the studies on the role of ceramides in the pathogenesis of psoriasis.

Author	Year	Population	Key Observation
Ceramides—An Overview			
Hannun et al. [1]	2018	Mammalian cells	Ceramides can be produced via the catabolism of sphingomyelin and glycosphingolipids, as well as via de novo biosynthesis starting from the formation of sphingoid bases and their subsequent N-acylation.
Myśliwiec et al. [4]	2017	n1- 85 patients with active plaque psoriasis, (19–79 years); n2- 32 healthy controls	A significantly higher percentage of MUFA and a lower percent of PUFA was demonstrated in both groups of psoriatic patients (with and without obesity) compared to the healthy control subjects.
Lew et al. [9]	2006	n- 5 patients with psoriasis, from 19 to 33 years	Decreased levels of ceramides have been reported in skin conditions involving dryness and barrier disruption, such as psoriasis.
Yokose et al. [10]	2020	Stratum corneum tape-stripping: n1 = 16, healthy control subjects; n2 = 10, PsO patients; n3 = 8, Cer profiles of AD patients	The level of Cer [NP] and the Cer [NP]/[NS] ratio are higher in the SC compared to KCs in normal human skin.
Lee et al. [11]	2009	Keratinocytes; epidermal growth factor, bovine pituitary extract; 5 μg of total RNA antibiotic-antimycotic	RA inhibits keratinocyte differentiation and lipid metabolism associated with the formation of epidermal barrier function.
Alessandrini et al. [12]	2004	n- 5 male subjects with psoriasis vulgaris; skin biopsies from lesional and non-lesional skin	The mRNA expression of GlcCer’ase is decreased in psoriatic epidermis; its level is higher in lesional compared with non-lesional psoriatic skin.

Abbreviation: Cer—ceramides; RA—retinoids; CPE—ceramide phosphoethanolamine; GE—Gentiana lutea extract; ELOVL—elongase; n1—healthy group; n, n2, n3—study group.

**Table 2:** Summary of the studies on sphingosine 1-phosphate (S1P) involvement in the pathogenesis of psoriasis.

Author	Year	Population	Key Observation
Sphingosine 1-phosphate (S1P) Involvement in Human Diseases			
Kozłowska et al. [2]	2019	n1- 32 healthy controls; n2- 85 patients with active plaque-type psoriasis.	The S1P concentration was higher in psoriatic patients with normal body weight and those overweight than in the control group. In psoriatic patients with normal body weight, nervonic ceramide (C24:1) correlated with PASI.
Jeon et al. [6]	2020	HEKn cells. Eight-week-old mice.	S1P is associated with inhibition of cell proliferation and induction of differentiation in human keratinocytes.
Moskot et al. [13]	2018	NPC1 mice. SPT-cKO mice.	S1P is responsible for angiogenesis, IL-17 uprising, Th17 cell development, T cell migration, and angiogenesis.
Sorokin et al. [14]	2018	n1- 30 healthy controls. n2- 60 psoriasis patients.	Decreased sphingosine-1-phosphate levels developed by excessive sphingosine-1-phosphate lyase activity, which may be associated with the keratinocyte hyperproliferation seen in psoriasis.
Ji et al. [15]	2018	n2- Female KM mice, 6–8 weeks old; n3- Female guinea pigs.	Syl930, which selectively modulates S1PR1, ameliorates acanthosis and parakeratosis.
Hong et al. [16]	2009	n- Adult female hairless mice, 8–10 weeks of age.	SphK1 activator K6PC-5 influences Ca2 signaling in human keratinocytes as well as epidermal differentiation and proliferation.
Schuster et al. [17]	2020	n- Mice were euthanized and skin samples harvested after 6, 24, or 72 h for further analysis.	Attenuated CCL2 production translates to reduced infiltration of Mφs into the inflamed tissue.
Setyawan et al. [18]	2021	n1- 182,431 patients, IMD cohort; n2- 182,431 patients, non-IMD cohort.	Sphingosine 1-phosphate receptor modulators were associated with decreased rates of TEs.
Rujimongkon et al. [19]	2021	n1 = 5; n2,3,4,5,6 = 5 each, rats with psoriasis.	Sericin reduced SPL expression in psoriatic skin.
Okura et al. [20]	2021	n- murine. Psoriasiform dermatitis was induced by imiquimod application.	Administration of fingolimod ameliorated IMQ-induced psoriatic dermatitis clinically and histologically.
Shin et al. [21]	2020	n- Eight-week-old male C57BL/6 mice.	HWG-35D significantly reduced thickness and scaling in patients with psoriasis.
Bocheńska et al. [22]	2019	HaCaT cell line.	Increased SPHK1 gene activity in psoriatic lesions, resulting in the production of tumor necrosis factor alpha.

Abbreviations: NKT—natural killer T cells; IMD—immune-mediated diseases; HEKn—human neonatal epidermal keratinocyte; SPT-cKO mice—serine palmitoyltransferase–conditional knockout; NPC1—Niemann–Pick disease type C; n1—healthy group; n, n2, n3, n4, n5—study groups.

**Table 3:** Summary of the studies on phospholipid metabolism in psoriasis.

Author	Year	Population	Key Observation
Phospholipid Metabolism in Psoriasis			
Shao et al. [23]	2021	n1 = 3; n2 = 22, PP; n3 = 13, PRP	Combination of TNF-α and IL-17A is a strong inducer of PLA2G2F, PLA2G4D, and PLA2G4E expression, and these PLA2s play a major role in the proinflammatory effects of IL-17A and TNF-α on the epidermis.
Li et al. [24]	2017	n1- 75 healthy volunteers; n2-75 patients with psoriasis vulgaris	Chenodeoxycholic acid in psoriasis vulgaris was lower than in healthy controls; leukotriene D4 and Llukotriene E3 in serum were significantly higher in psoriasis patients.
Farwan-ah et al. [25]	2005	n1- 7 patients; n2- 7 patients with AD; n3- 6 patients with PP	The ceramide amount in the uninvolved skin of atopic dermatitis (15.3 ± 4.0 μg/cm^2^) and psoriasis (11.4 ± 3.5 μg/cm^2^) patients is reduced when compared to healthy skin (16.2 ± 3.5 μg/cm^2^).
Moon et al. [26]	2013	n- 8 patients with psoriasis vulgaris	The sphingosine and sphinganine amounts are significantly elevated in psoriatic epidermis compared to non-lesional epidermis.
Łuczaj et al. [27]	2020	n1- 6 patients; n2- 6, psoriasis vulgaris	The levels of several species of different classes of phospholipids, including PC, PI, PS, and PEo, are reduced in the keratinocytes of patients with psoriasis.
Zeng et al. [28]	2017	n1- 45 patients; n2- 45, psoriasis	LPA and PA are dramatically increased in psoriasis patients compared to healthy controls.
Kim D. et al. [29]	2021	Six-week-old male Balb/c mice; n1- mice; n2- mice, IMQ- vehicle-treated group; n3- mice, IMQ- ki16425-treated group	LPA induced ROCK2 and PI3K/AKT signaling pathway-mediated cell cycle transformation and proliferation in keratinocytes.
Pang et al. [30]	2015	n1- 84 patients; n2- 86 patients with psoriasis	The fasting serum values of TC, HDL-C, LDL-C, and ApoA-I for the patient group were all significantly lower than those in healthy controls. The levels of fasting serum TG and ApoB_100_ did not show any significant difference between the patient group and healthy controls.
Ferretti et al. [31]	2012	n1- 25 patients; n2- 23 patients with psoriasis	Higher levels of lipid hydroperoxides and lower PON1 activity were observed in the serum of patients. Higher levels of Lp(a) in the serum of patients with psoriasis compared with controls.
Tyrrell et al. [32]	2021	n1- 5 patients; n2- 9 patients with psoriasis	ZIC1 may play a central role in upregulating their metabolism in psoriasis.
Gaire et al. [34]	2020	n- Male BALB/c mice, 6 weeks old, Orient Bio, Gyeonggi-do, Korea	Suppressing LPA_5_ activity with a pharmacological antagonist alleviated IMQ-induced psoriasis-like symptoms.
Yamamoto et al. [33]	2016	n- Mice (BALB/c background, 8–12-week-old males)	In psoriasis and skin cancer, sPLA_2_-IIF is upregulated in the thickened epidermis and promotes epidermal hyperplasia through production of the unique P-LPE.
Syed et al. [35]	2020	n- Mice, primary human monocytes from healthy donors	Direct involvement of SPHKs in macrophage NLRP3 inflammasome activation provides a rationale for therapeutic targeting of these kinases in cancer and inflammation.
Ogawa et al. [36]	2011	n- E-FABP-/- mice	E-FABP affects keratinocyte differentiation, which suggests that E-FABP may have a role in the pathogenesis of psoriasis.
Shou et al. [37]	2021	n1- 10 patients; n2- 8 patients with psoriasis; n3- female BALB/c-Mice at 6–8 weeks of age	The pathogenic role of ferroptosis in sensitizing Th22/Th17-type cytokines was described, thereby providing candidate markers of psoriasis aggravation.
Utsunomiya et al. [44]	2020	n2- DMKN-βγ-/-mice; n3- DMKN-αβγ-/- mice	Modestly reduced levels of some ceramide species, including EOS (a representative acylCer), were consistent with mild skin barrier dysfunction in DMKN-αβγ-/- mice.

Abbreviations: IMQ-Ki—IMQ with ki16425-treated group; IMQ-Veh—IMQ with vehicle-treated group; n1—healthy group; n, n2, n3, n4, n5—study groups.

**Table 4:** Summary of the studies on sphingolipid metabolism in psoriasis.

Author	Year	Population	Key Observation
Psoriasis			
Kozłowska et al. [2]	2019	n1- 32 patients; n2- 85 patients with psoriasis	S1P concentration was higher in psoriatic patients with normal body weight and those overweight than in the control group. In psoriatic patients with normal body weight, nervonic ceramide (C24:1) correlated with PASI.
Łuczaj et al. [3]	2020	n1- 6 patients; n2- 6 patients with psoriasis vulgaris	The results showed higher levels of CER[NS], CER[NP], CER[AS], CER[ADS], CER[AP], and CER[EOS] in the keratinocytes of patients with psoriasis, while the CER[NDS] level was lowered compared to the control samples.
Moskot et al. [13]	2018	n2- NPC1 mice; n3- SPT-cKO mice	S1P is responsible for angiogenesis, IL-17 uprising, Th17 cell development, T cell migration, and angiogenesis.
Cho et al. [48]	2004	n- 10 patients with plaque-type psoriasis	The amount of ceramides in the human epidermis is controlled by a stability in the activity of ceramide generating enzymes (for instance β-glucocerebrosidase, serine palmitoyl transferase, sphingomyelinase) and the degradative enzyme (for example ceramidase). Ceramides in the stratum corneum are originally descended from sphingomyelin and β-glucosylceramides.
Holleran et al. [38]	2016	Mammalian skin; FAN-deficient mice; prosaposin-deficient mice	The enzymatic transformation of glucosylceramides to ceramides may be disturbed by reduced levels of prosaposin and saposin in psoriatic skin.
Checa et al. [5]	2017	n1- 32 healthy patients; n2- 32 patients with mild psoriasis; n3- 32 patients with severe psoriasis	Ceramides with chain lengths of 16–24 were higher in severe patients with psoriasis. Circulating levels of the C_12:0_-ceramide were significantly decreased in severe psoriasis and treatment restored levels to normalcy.
Gao et al. [42]	2021	n- 20 BALB/C male mice (8 weeks old)	A successful knockdown of the PLA2G4B gene by siRNA-505 candidates.
Li et al. [24]	2017	n1- 75 healthy patients; n2- 75 patients with psoriasis vulgaris	Chenodeoxycholic acid in psoriasis vulgaris was lower than in healthy controls; leukotriene D4 and leukotriene E3 in serum were significantly higher in psoriasis patients.
Souto-Carneiro et al. [41]	2020	n2- 49 patients with negRA; n3- 73 with PsA	Lipid β-methylenes and lipid α-methylenes can mostly be associated with changes in the levels of lipids in the serum.
Myśliwiec et al. [40]	2019	n1- 32 healthy patients; n2- 54 active plaque-type psoriasis; n2a- 40 without PsA; n2b- 14 with PsA	An abnormal FA profile with a higher SFA/UFA ratio in PsA compared to psoriasis without arthritis; lower concentration of PUFA and higher concentration of MUFA in psoriasis and PsA compared to healthy control.
Del Rosso et al. [45]	2017	n- 33 with a history of mild-to-moderate psoriasis	In this study, application of a ceramide/keratolytic-containing cream showed benefits within two weeks in patients with psoriasis.
Choi et al. [46]	2005	Comparison of the lipid structure of the skin in atopic dermatitis, psoriasis, and healthy controls	A major alteration to ceramide composition in psoriasis was associated with a significant decrease in the percentage of phytosphingosine-carrying ceramides.
Cho et al. [48]	2004	n- 10 psoriatic patients	The level of ceramide synthesis is significantly reduced in the lesional epidermis, which is sensitive enough to make an inverse correlation with clinical severity in mild to moderate status psoriasis.
Tessema et al. [49]	2018	Soybean GlcCER (> 99% by TLC)	ME was shown to improve the penetration of oat CERs in vitro as well as ex vivo compared to the other formulations. On the other hand, the NPs sustained the release of oat CERs.
Egger et al. [50]	2020	Patients with psoriasis and without psoriasis; Cav1-null mice	There was a significant reduction in Cav1 expression in lesional skin compared to non-lesional skin.
Geilen et al. [51]	1996	HaCaT cells, human keratynocytes	The known inducers of SM hydrolysis, 1 a,25_dihydroxyvitamin D and TNFa, both responded to the antiproliferative, synthetic vitamin D analog, calcipotriol.
Zhang et al. [52]	2018	Glut1fl/fl mice	GLUT1 was also essential for proliferation in human keratinocytes in vitro.
Li et al. [53]	2020	n- 178 patients with mild-to-moderate psoriasis vulgaris	LA-Cer moisturizer alleviated psoriasis by maintaining and prolonging the improvement achieved by topical GC treatment.
Liu et al. [54]	2020	8–11 weeks-old BALB/c female mice weighing 20–25 g	Demonstrates a significant anti-psoriasis effect by (R)-salbutamol.
Simoni et al. [55]	2013	Mice- no data available, patients with psoriasis, MS, SLE, RA	CD4^+^ iNK T cells infiltrate lesions in psoriasis and atherosclerosis and might be pathogenic.
Breiden et al. [56]	2014	Mice- no data available	Transepidermal water loss is due to the drastically reduced content of total linoleic acid and acylceramides.
Motta et al. [47]	1994	n1- 6 healthy patients; n2- 6 psoriatic patients	In all types of psoriatic scale, CER 1, 3, 4, 5n, and 6 content was decreased, while CER 2h 2n, and 5 content was increased.
Hong et al. [57]	2007	n- 24 patients with psoriasis	Levels of SPT expression in the lesional epidermis were significantly lower than those in the non-lesional epidermis.
Tawada et al. [43]	2014	n1- 22 healthy patients; n2- 15 psoriatic patients; n3- 15 with AD patients	IFN-γ downregulates ELOVL1 and CERS3, which in turn reduces the levels of CERs with long-chain fatty acids in human skin.

Abbreviations: SPT-cKO mice—serine palmitoyltransferase–conditional knockout; NPC1—Niemann–Pick disease type C; FAN—factor associated with neutral sphingomyelinase activity-deficient mice; n1—healthy group; n, n2, n2a, n2b, n3, n4, n5—study groups.

**Table 5:** Summary of the studies on sphingolipids in psoriasis and obesity and metabolic syndrome.

Author	Year	Population	Key Observation
Obesity			
Kozłowska et al. [2]	2019	n1- 32 healthy patients; n2- 85 psoriatic patients	In overweight patients, the concentration of lignoceric ceramide (C24:0) correlated inversely with the severity of the disease.
Kozłowska et al. [58]	2021	n1- 32 healthy patients; n2- 85 psoriatic patients	The reduction in C24:0 was linked with increased insulin sensitivity and weight reduction. In young obese adults, a positive link was observed between γ-glutamyl transpeptidase and C24. The levels of C14:0, C16:0, C22:0, and C24:0 were elevated in prediabetic and diabetic nonhuman primates, and the levels of these ceramides correlate with the HOMA-R.
Majumdar et al. [59]	2012	n1- 15 healthy patients; n2- 30 psoriatic patients	A high level of glucose induces Sphk in vascular endothelial cells in vitro and leads to synthesis of S1P, which subsequently activates vascular endothelial cell growth.

Abbreviations: n1—healthy group; n2—study group.

**Table 6:** Summary of the studies on sphingolipid metabolism in psoriasis and liver disease.

Author	Year	Population	Key Observation
Liver Diseases			
Kozłowska et al. [58]	2021	n1- 32 healthy patients; n2- 85 psoriatic patients	Lignoceric ceramide positively correlated with ALT level in psoriatic patients.

Abbreviations: n1—healthy group; n2—study group.

**Table 7:** Summary of the studies on sphingolipids in psoriasis and cardiovascular diseases.

Author	Year	Population	Key Observation
Heart Diseases			
Hadas et al. [60]	2020	CFW male and female mice, modRNA, sphingolipids	AC overexpression is sufficient and necessary to induce cardioprotection post MI.
Poss et al. [61]	2020	n1- 212 healthy patients; n2- 462 individuals with familial CAD	Cer (18:1/24:0) is a good marker of CAD. Ceramide concentrations were shown to be elevated among individuals with heart events.
Kovilakath [62]	2020	Mice, zebrafish	There were increased plasma concentrations of C16:0 and C18:0 ceramides in participants with HFpEF.
Altekin et al. [63]	2012	n1- 60 healthy patients; n2- 75 plaque-type psoriasis patients	PWV and the average and maximum CIMT values of psoriasis patients were higher than in the healthy group.
Wang et al. [39]	2022	n2- 136 PsA patients; n3- 152 PsO patients	PsA patients had a higher ApoB/ApoA1 ratio than PsO patients. ApoB was positively associated with concomitant arthritis, diabetes, and hypertension.
Ilanbey et al. [64]	2021	n1- 52 healthy patients; n2- 53 psoriatic patients	ChT activity was found to be higher in patients with hypertension than in those with other comorbidities.
Lasa et al. [65]	2021	n- 9604 patients who were exposed to an S1P modulator	Transient cardiovascular events (such as bradycardia and AV block) are increased with S1P modulators.

Abbreviation: ApoA1—apolipoprotein A1; ApoB—apolipoprotein B; CFW—Swiss Webster; HF—heart failure; n1—healthy group; n, n2, n3—study groups.
